# Enhancement of Immune Activation Activities of *Spirulina maxima* Grown in Deep-Sea Water

**DOI:** 10.3390/ijms140612205

**Published:** 2013-06-06

**Authors:** Woon Yong Choi, Do Hyung Kang, Hyeon Yong Lee

**Affiliations:** 1 Department of Medical Biomaterials Engineering, Kangwon National University, Chuncheon 200-701, Korea; E-Mail: ahdcl1012@gmail.com; 2 Korea Institute of Ocean Science and Technology (KIOST), Ansan 426-744, Korea; E-Mail: dohkang@kordi.re.kr; 3 Department of Food Science and Engineering, Seowon University, Cheongju, Chungbuk 361-742, Korea

**Keywords:** β-carotene, ascorbic acid, deep-sea water, immune activity, *Spirulina maxima*

## Abstract

In this study, the immuno-modulatory and anticancer activities of marine algae, *Spirulina maxima* grown in deep-sea water (DSW), were investigated. It was found that the extract of *S. maxima*, cultured in DSW, effectively suppressed the expression of Bcl2 in A549 cells as well as inhibiting various human cancer cells with concentration dependency, which possibly implies that the extracts may play more important roles in controlling cancer cell growth. The secretion of cytokines IL-6 and TNF-α from human B cells was also greatly increased, compared to those of the extract grown in conventional sea-water. The growth of Human Natural Killer (NK) cells in the presence of the extracts from DSW was significantly higher (12.2 × 10^4^ viable cells/mL) when compared to the control (1.1 × 10^4^ viable cells/mL). Based on HPLC analysis, the increase in the biological activities of the extracts from DSW was caused by considerably high amounts of β-carotene and ascorbic acid because the DSW contained high concentrations and good ratios of several key minerals for biosynthesizing β-carotene and ascorbic acid, as well as maintaining high cell growth.

## 1. Introduction

*Spirulina* sp. is 300 μm to 500 μm long and eight meters in diameter, and lives in salt lakes in tropical regions [1]. About 35 different strains of *Spirulina* have been identified, among them, the cyanobacterium, *Spirulina maxima*, inhabits carbonate-rich lakes in torrid zones [2]. Many studies have examined the effects of *Spirulina* on modulation of the host immune system, and their anti-cancer activities [3–8] because *Spirulina platensis* contains high quality proteins, iron, gamma-linoleic fatty acids, carotenoids, and vitamins, *etc.* [1,9]. Due to its unique structural properties, lack of cellulose, low nucleic acid content, 56% protein content, 1% fat content, 16% carbohydrate content, and large mineral, vitamin, fiber, and pigment content, *S. platensis* is easily digested and absorbed in the body [1]. Many researchers have examined the efficacy of *S. platensis* in enhancing fat digestion and inhibiting lipid oxidation due to its phycocyanin, vitamin B12, phenoic acid, and tocopherol content [10,11]. In addition, it has been reported that carotenoid, phycocyanin, and novel sulfated polysaccharide, display anti-aging, antitumor, and antioxidant activities [12–15]. Therefore, *Spirulina* has been widely used as food and nutritional health supplements in over 100 countries, and its beneficial health effects have been recognized by several international organizations including WHO, FAO, UNIDO, and UNICEF [16].

Compared to *S. platensis*, however, relatively few studies have examined the biological activities of *S. maxima*, even though it has a number of advantages, including its easy scale-up due to its fast cell growth, and rich protein and essential amino acid content, which are comparable to *S. platensis* [1]. Therefore, it is necessary to develop the proper cultivation conditions for the large-scale production of *S. maxima* biomass. With this goal in mind, we examined the potential of using deep-sea water as a culture medium to grow *S. maxima*. Deep-sea water has a relatively constant temperature, abundant nutrients such as calcium, magnesium, nitrates, and phosphates, *etc*., and stable water quality, even though there might be some variations of their compositions according to collection places [16–23]. Thus, deep-sea water would be a good substrate for algal growth and biomass production since it contains various nutrients, including a fluorescent red pigment, and β-carotene, *etc.* [1,24]. Therefore, the objective of this study was to investigate the potential of using deep-sea water to enhance the anticancer and immuno-modulatory activities of *S. maxima* through a relatively simple and cheap medium, compared with those grown in conventional sea-water enforced with essential nutritive medium.

## 2. Results and Discussion

### 2.1. Measurement of Cytotoxicity and Anticancer Activity

**Figure 1:**
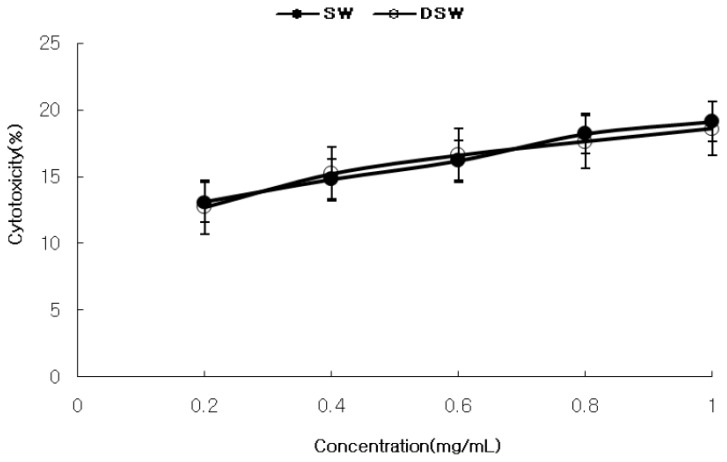
Cytotoxicity of the extracts of *S. maxima* cultivated in SW and DSW on the normal cell line, HEK 293. SW and DSW extracts were extracted at 100 °C. Mean ± SD from triplicate separated experiments are shown. (SW: seawater, DSW: deep-sea water).

**Figure 2:**
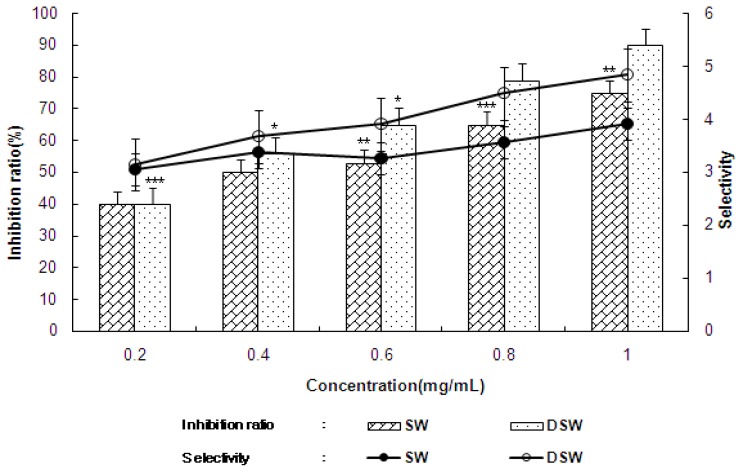
Inhibition of human stomach adenocarcinoma (AGS) (bar chart, percent) and selectivity (line chart) after adding 1.0 mg/mL of the extract of *S. maxima* cultivated in SW and DSW. Mean ± S.D. from triplicate separated experiments are shown. Each value were compared with the control at * *p* < 0.01, ** *p* < 0.005, *** *p* < 0.001 by Student *t*-test. (SW: seawater, DSW: deep-sea water).

Figure 1 shows the cytotoxicity of *S. maxima* against human normal kidney cells, which increased in a concentration dependent manner (0.2~1.0 mg/mL). The water extracts from seawater (SW) had a higher cytotoxicity (19.1%) than the water extract from the deep-sea water (DSW) (17.5%) at the highest extract concentration (1.0 mg/mL). As shown in Table 1 and Figure 2, DSW showed a stronger cytotoxicity against most cancer cell lines than SW, as well as a higher selectivity that implies the ratio of selectively inhibiting the cancer cells compared to normal cell. Interestingly, DSW showed a stronger inhibition (85%–93%) at higher concentrations than SW at concentrations lower than 0.4 mg/mL for most cancer cells, especially in MCF-7 and Hep3B, respectively. It is worth noting that this inhibition level was similar to, or even higher than, those reported for the addition of *S. plantesis* extracts [5,25], this result of depressing various cancer cells implies that the extracts could play a role in inhibiting cancer cell growth by improving immune activation [26]. Therefore, the results of investigating immune activation activities of DSW extracts were shown for the following experiments.

### 2.2. Measurement of Bcl-2 Expression in Human Cancer Cell

**Figure 3:**
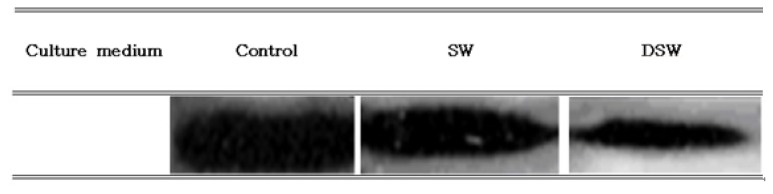
The expression of Bcl-2 in A549 cells after adding 1.0 mg/mL of the extract of *S. maxima* grown in different media. (SW: seawater, DSW: deep-sea water).

To examine the anticancer activities of the samples at the molecular level, the downregulation of Bcl-2 expression in A549 cells was determined since A549 cell growth was also similarly inhibited in treating the DSW extract (data not shown). Bcl-2 was selected for this purpose because it is an anti-apoptosis gene that indirectly inhibits apoptosis by inhibiting the opening of PT channels on the mitochondrion, which lowers the mitochondrial potential and blocks the release of cytochrome C from the mitochondrion [27]. As shown in Figure 3, the control, which did not contain any samples, had a higher level of Bcl-2 protein expression than the two other cases, and the Bcl-2 expression level was lowest in cell treated with 1.0 mg/mL of DSW. This result clearly indicates that the extract from DSW could effectively inhibit the proliferation of cancer cell growth by reducing the expression of Bcl-2 in human stomach cancer cell, which was well correlated to the results shown in Figure 3.

### 2.3. Secretion of Cytokine, Interleukin-6 (IL-6), and Tumor Nucreosis Factor-α (TNF-α) from the Growth of Human B and T Cells

**Figure 4:**
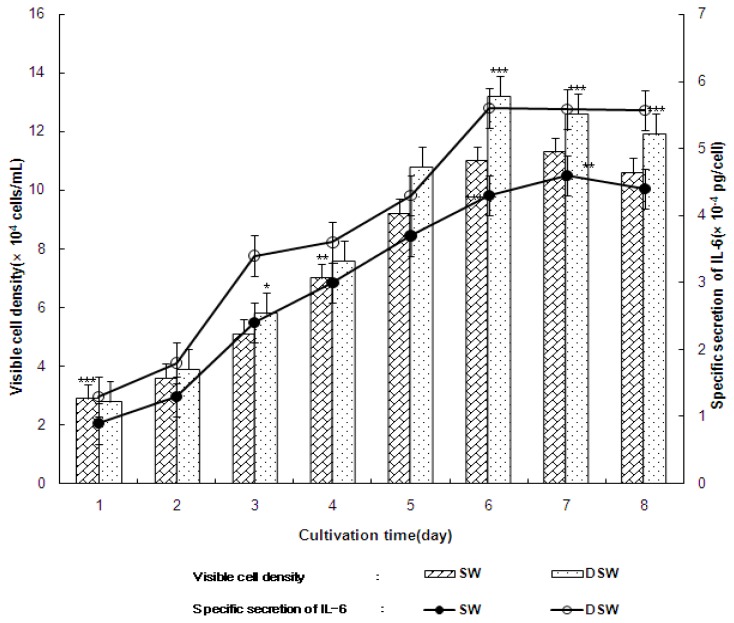
The growth (bar chart, percent) and secretion (line chart) of Interleukin-6 (IL-6) from human B cell after the adding 1.0 mg/mL of the water extract of *S. maxima* cultivated in SW and DSW. Mean ± S.D. from triplicate separated experiments are shown. Each value were compared with the control at * *p* < 0.01, ** *p* < 0.005, *** *p* < 0.001 by Student *t*-test. (SW: seawater, DSW: deep-sea water).

The above data demonstrated that the extracts from DSW had strong anticancer activities, which indicates that they may also have immuno-modulatory activities since many studies have reported that there is a close relationship between anticancer and immune activation activities in natural extracts [28,29]. In Figure 4, the growth of human B cell and secretion of Interleukin-6 (Il-6) was compared between the addition of 1.0 (mg/mL) of SW or DSW. In these experiments, it was apparent that the extract from deep-sea water enhanced both cell growth and secretion of IL-6 from B cells. In addition, cell growth was maintained during the latter periods of the cultivation in the presence of DSW, while cell growth decreased in the presence of SW. This may have been the reason why the extract obtained from deep-sea water could improve cell growth, which also resulted in the constant specific production of IL-6, even during the latter period of cultivation. Similar results were also observed for natural products that displayed immune activation activity since the secretion of specific cytokines, such as IL-6 or Tumor Nucreosis Factor-α (TNF-α), was closely related to immune-modulatory activities within the cells [1,30]. Table 2 also clearly demonstrates that DSW enhanced immune functions by increasing both cell growth and IL-6 and TNF-α secretion, compared to the addition of SW. It should also be noted that the extracts had a stronger positive effect on T cell growth than B cell growth. As shown in Table 2, cell growth after the addition of the *S. maxima* extract continually increased from one to six days. Particularly, the growth of human T cells increased by over 2.4 × 10^5^ viable cells/mL in the presence of 1.0 mg/mL of DSW. In general, T cell growth (24 × 10^4^ viable cells/mL) in the presence of *S. maxima* cultivated in deep-sea water was significantly higher than that of SW (6.4 × 10^4^ viable cells/mL). The DSW also increased B cell growth (18 × 10^4^ viable cells/mL) compared to seawater culture mediums. The secretion of interleukin-6 and TNF-α by human T cells were also increased up to over 5.30 × 10^−4^ pg/cell and 5.60 × 10^−4^ pg/cell, respectively through the addition DSW. The secretion of IL-6 in the presence of deep-sea water was higher than IL-6 secretion in the presence of seawater (Table 2). A similar pattern as seen with the cytokine secretion from T cells was also observed for B cells, where the secretion of Interleukin-6 and TNF-α were increased to over 5.30 × 10^−4^ pg/cell and 5.60 × 10^−4^ pg/cell, respectively, through the addition DSW.

### 2.4. Enhancement of NK Cells Associated with Human T Cell Growth

**Figure 5:**
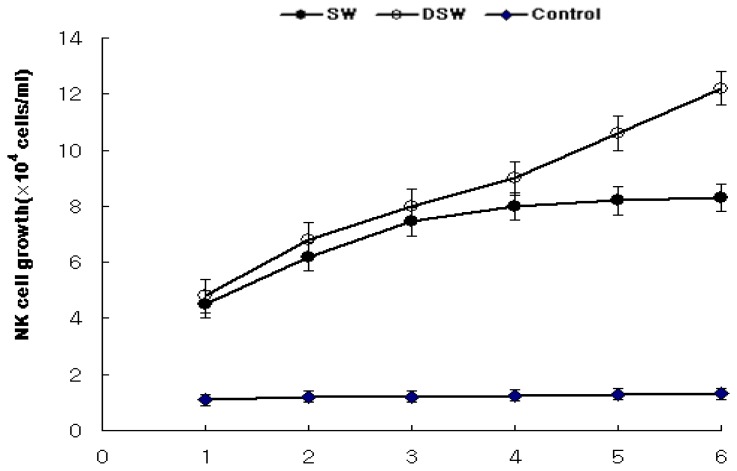
The NK cell growth after the addition of supernatants from T cells that were treated with the water extract of *S. maxima* cultivated in SW and DSW. Mean ± S.D. from triplicate separated experiments are shown. (SW: seawater, DSW: deep-sea water).

In addition to measuring the increase in the secretion of cytokines from B or T cells, the immune activation activities of the extracts must also be confirmed by observing NK cell growth after the addition of stimulated B and T cells since the cytokines released from these cells could affect NK-92MI cell growth promotion [31,32]. In general, NK cell growth gradually increased according to the cultivation time after the addition of the samples, as shown in Figure 5. The extracts of DSW significantly increased NK cell growth to over 12.2 × 10^4^ viable cells/mL, while the extracts of SW only increased cell growth by approximately 8.3 × 10^4^ viable cells/mL. The increase in NK cell growth during the latter periods of cultivation, in the presence of the seawater extract, was significantly lower than in the presence of the deep-sea water extract. However, all the extracts of *S. maxima* appeared to improve NK cell growth relative to the control (not adding any extract), which could not maintain the growth of NK cells. This result clearly demonstrates that NK cell growth was significantly promoted due to the high amount of cytokines released after the addition of the deep-sea water *S. maxima* extract, relative to the extract of *S. maxima* cultivated in a seawater medium.

### 2.5. HPLC Analysis

**Figure 6:**
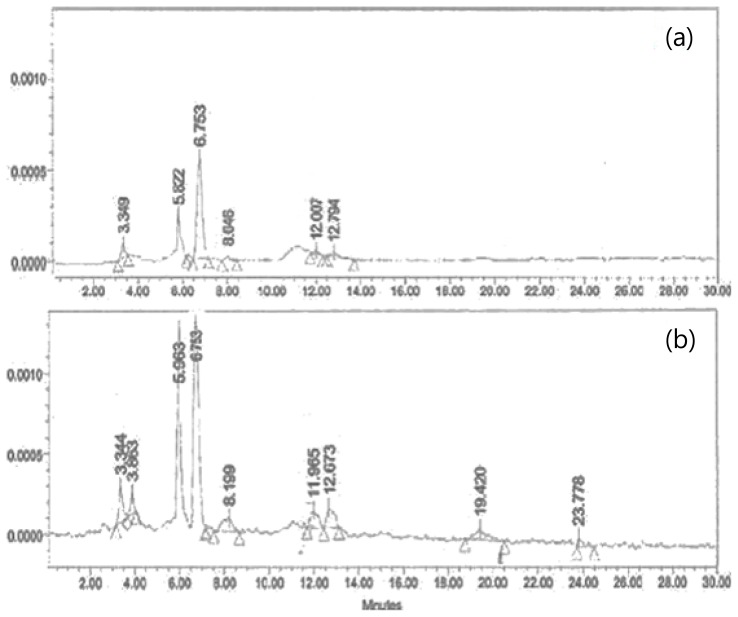
Comparison of the HPLC chromatograms for the water extract of *S. maxima* cultivated in seawater (**a**) and deep-sea water (**b**).

The results presented above indicate that the *S. maxima* extracts, grown from deep-sea water, had a stronger physiological effect than the extracts grown in seawater, particularly in regards to the immune activation activities associated with anticancer activities. To better understand the biological activities of the extracts, the HPLC profiles of the extracts grown in the two different medium was determined to identify the key biologically active components in *S. maxima* (Figure 6). This was done because previous studies have reported that *S. maxima* contains a high content of β-carotene, ascorbic acid, thiamin, and γ-linolenic acid, iron *etc.*, all of which have strong anticancer and immune activities [33–37]. Particularly, β-carotene and vitamin C have multiple biological activities such as anticarcinogenic [33,34], antimutagenic [35], antioxidant, anti-inflamnatory, antiprolifertive [33], and antiatherogenic properties [38]. In addition, these compounds have been used as chemo-preventive agents against cancer diseases in various organs such as the lung, stomach, colon, breast, and prostate [37]. The extract cultivated under deep-sea water conditions had higher amounts of all the components than the extracts cultivated in seawater (SW). Using the absorbance of the standard compounds at 254 nm, the *S. maxima* extracts grown in deep-sea water (DSW) were found to contain 1100 μg/g of β-Carotene (peak retention time six minutes as a standard), 12.4 μg/g of ascorbic acid (peak retention time five minutes) and 14 μg/g of thiamin (peak retention time three minutes). These concentrations were higher than those grown in conventional seawater (520 μg/g of β-Carotene, 7.3 μg/g of ascorbic acid and 8 μg/g of thiamin). Moreover, in comparing these values to those reported in other studies (800 μg/g of β-Carotene, 6.7 μg/g of ascorbic acid, and 10 μg/g of thiamin) [39], it appears that the deep-sea water improved the biosynthesis of all three components and the extracts cultivated in seawater only displayed an increase in the ascorbic acid concentration. From various reported data, 2500 ug/g of pure carotene or 60 ug/g of ascorbic acid inhibited cancer cell lines and also enhanced immune activation activities by improving 20% [26,40]: however, the efficacy of each single compound was somewhat lower than those of the extracts containing both compounds, possibly by synergic activities between them, as well high treatment concentrations. Therefore, these results clearly indicate why the DSW extract displayed a higher immune activation activity and a higher anticancer activity [28,29].

### 2.6. Discussion

There have been several reports on the biological activities of *S. platensis*; however, there have only been a comparably small number of studies on the biological activities of *S. maxima*. In addition, the effects of cultivation in deep-sea water on the biological activities of the extracts have received little attention, even though deep-sea water is a clean resource that contains high concentrations of several important minerals for micro algal cultivations. There have been several studies that reported an increase in cell growth and better physiological performance when samples were cultivated in deep-sea water [18,41,42], however, an increase in the biological functions of the extracts after cultivation in deep-sea water has not been examined, which could explain why deep-sea water increases the immune activation activities of *S. maxima*.

Therefore, in this work, the effect of a higher content of β-Carotene and ascorbic acid on the biological activity of the extract was examined by growing them in deep-sea water. This was done because these compounds are known to play an important role in multiple biological activities such as anticarcinogenic [33,34,43], antimutagenic [35], antioxidant [36], and anti-inflamnatory activity, *etc.*, In general, the extracts grown from deep-sea water (DSW) had less cytotoxicity against normal human cell lines than the extracts cultivated in conventional seawater (SW) (19% *vs.* 23%). This result was comparable to the results reported for the *S. platensis* extract, which had a cytotoxicity of approximately 26% [5]. The extracts inhibited cancer cell growth in the range of 75% to 91%, after the addition of 1.0 mg/mL of the extract. This was in good agreement with a previous study, which reported that the *S. platensis* extracts inhibited the growth of cancer cells by more than 75% [5]. The anticancer activities of the extracts may have been caused by an increase in human immune cell growth associated with the increase of cytokines such as tumor necrosis factor (TNF)-α and IL-2 because tumor cells can acquire resistance to (TNF)-α related apoptosis-inducing ligand (TRAIL), an innate immune molecule that selectively induces apoptosis in tumor cells [44,45]. The results of our work indicate that DSW extracts may enhance TRAIL-induced apoptosis, which can sensitize several cancer cells [46–51]. This hypothesis was also supported by the result that Bcl-2 expression level was significantly decreased in the presence of the DSW extracts. Overall, the extracts obtained from deep-sea water displayed stronger immuno-modulatory activities than the extracts from seawater, which also resulted in higher anticancer activities because of the significantly higher β-Carotene and ascorbic acid contents of the extracts grown in deep-sea water.

It was first reported that *S. maxima* grown in deep-sea water contained high amounts of β-carotene and ascorbic acid because deep-sea water has high calcium and magnesium contents, as well as nitrate and phosphate contents [41]. Because of this higher nutritive mineral content, cell growth was activated, which could result in a higher β-carotene and vitamin C content [41,42]. Deep-sea water is used in numerous fields ranging from foods to medicines, and health products, and the results of this study indicate that deep-sea water could be used to develop bioactive substances from *S. maxima*. However, further studies on identifying other new active components in DSW and more detailed immune activation mechanisms should be carried out for widely expanding the use of its extracts.

## 3. Experimental Section

### 3.1. Cells and Chemicals

The strain of *S. maxima* (CY-023) was obtained from Korea Marine Microalgae Culture Center (Pukyoung National University, Pusan, Korea), and all cell lines (human breast adenocarcinoma MCF-7, human gastric cancer cell line AGS, human hepatocillular carcinoma Hep3B, human embryonic kidney cell HEK 293, human lung carcinoma A549, NK cell and human T cell, B cell) were obtained from American Types of Culture Collection (ATCC, Manassas, VA, USA). RPMI 1640 and alpha minimum essential medium (α-MEM) were purchased from GIBCO (Invitrogen, Carlsbad, CA, USA). HEPES buffer was purchased from SIGMA (St. Louis, MO, USA). Fetal bovine serum and horse serum was purchased from GIBCO (Invitrogen). Gentamycin sulfate and sulforthodamine B (SRB) were purchased from Sigma. Deep-sea water was collected at a 300 m-depth from the sea surface at the Deep Ocean Water Application Research Center of the Korea Ocean Research & Development Institute (Goseong, Korea). The concentrations of key minerals in the seawater (SW) and the deep-sea water (DSW) are compared in Table 3.

### 3.2. Culture Condition and Extraction of *S. maxima*

*S. maxima* was cultivated in two types of media: Seawater medium containing NaHCO_3_, K_2_HPO_4_, NaNO_3_, K_2_SO_4_, NaCl, MgSO_4_·7H_2_O, CaCl_2_, FeSO_4_·7H_2_O, EDTA, Solution A5 (A5 contained: H_3_BO_3_, MnCl_2_, ZnSO_4_, CUSO_4_, MoO_2_), and Solution B6 (NH4VO_3_, K_2_Cr(SO_4_)_4_, NiSO_4_, Na_2_WO_4_, Ti(SO_4_)_3_, Co(NO_3_)_2_), and Deep sea water medium containing only NaHCO_3_ as an inorganic carbon source [52]. Five percent (*w*/*v*) of the cells were grown in a 1 L Erlenmeyer flask (working volume 500 mL) for batch cultivation at 28 ± 2 °C, and continuously illuminated with white fluorescent lamps (40 W). Air containing 0.3% CO_2_ (*v*/*v*) was constantly supplied at a rate of 250 (mL/min) and cells were cultivated at an agitation speed of 150 rpm [52,53]. The cells were then collected by centrifuge at 3000 rpm from a culture flask. The collected cells were dried by ambient air at 20 °C before extraction. The powdered samples of *S. maxima* were extracted with water at 100 °C for 12 h. The water extract was concentrated in a rotary vacuum evaporator (Eyela, Tokyo, Japan), then freeze dried, and stored at −20 °C before use.

### 3.3. Measurement of Cytotoxicity and Anticancer Activity

*In vitro* cytotoxicity against several human cancer cell lines including MCF-7, Hep-3B, AGS, and normal human embryonic kidney cell (HEK 293) was measured using the modified Sulforhodamine B (SRB) assay [5,54] as follows: All cell lines were incubated in tissue culture flasks in the desired media with 5% CO_2_ at 37 °C. Trypsinized (trypsin-EDTA, Gibco (invitrogen, Carlsbad, CA, USA)) cell cultures were washed with media and diluted to 1 × 10_4_ viable cells/mL in each well of a 96 well plate. The plates were incubated at 37 °C for 48 h with the addition of several concentrations of the extracts. A hundred microliters of 20% ice-cold trichloroacetic acid (TCA) solution was then gently layered on top of the medium overlaying the cells. The plates were incubated for 60 min at 4 °C. The plates were rinsed several times with tap water and then the cells were stained with a 0.4% SRB solution (50 μL stain/well) for 15 min at room temperature. The SRB staining solution was removed, wells were then rinsed five times with 1% acetic acid to remove unbound dye, and left to air-dry. The bound SRB dye was solubilized by adding unbuffered Tris-base solution (100 μL/well), and the plates were placed on a plate shaker for one hour at room temperature. Plates were then read at 540 nm, using a microplate reader (Thermo max, Molecular Devices, Sunnyvale, CA, USA) and the results were expressed as the percentage of the control values.

The selectivity of extracts on each cancer cell line was estimated using the following equation;

(1) Selectivity=AA/CT

where AA and CT indicate the anticancer activity ratio and the extract-induced cytotoxicity on HEK 293 cells, respectively.

### 3.4. Measurement of Bcl2 Protein Level

The expression levels of Bcl-2 in AGS cells cultivated with, and without, the samples (control) were measured as follows [55]. The cells were lysed directly into a solution containing 1% Triton X-100, 20 mM Tris (pH 8.0), 137 mM NaCl, 15% glycerol, 5 mM EDTA, 1 mM Pefablock, 0.1 mg/mL aprotinin, and 0.1 mg/mL leupeptin. The debris was removed by centrifugation for 15 min at 15,000 × *g* and 4 °C, and equivalent amounts of protein were separated by 10% SDS-PAGE [56,57] and transferred onto nitrocellulose filters. The filters were firstly stained to confirm uniform transfer of all samples, and then incubated in blocking solution for two hours at room temperature. The filters were reacted first with the anti-Bcl-2 at a dilution of 1:1000 for two hours, followed by two washes with PBS and two washes with TBST. Filters were then incubated with 1:1000 horseradish peroxidase-conjugated anti mouse secondary antibodies for one hour, then washed with TBST and developed using the Super Signal West Pico Kit.

### 3.5. Measurement of Human T and B Cell Growth and Secretion of Cytokines

Human immune T cell (Jurkat; ATCC) and B cell (Raji; ATCC) cells were cultured at 37 °C in RPMI 1640 medium containing 10% FBS in 5% CO_2_. Each sample of 0.5 g/L was added and cell growth was measured by the number of cells in 24 well plates that initially contained 1.0 × 10^4^ viable cells/mL using trypan dye in a hemacytometer [58]. Secretion of cytokines, IL-6 and TNF-α were also quantified using IL-6 and TNF-α kits (Chemicon, Billerica, MA, USA) as follows [59]. After adjusting the cell concentration to 1~2 × 10^4^ viable cells/mL, 900 L the cell concentrate was placed into 24 well plates and cultured for 24 h (37 °C, 5% CO_2_). 100 L of the cells with 0.5 g/L extract was cultured again. The cell cultures were centrifuged to obtain the supernatant and the absorbance of the supernatant at 450 nm was read using a microplate reader (Thermo max, Molecular Devices). The amounts of cytokines were measured using the O.D. values of the standards.

### 3.6. Enhancement of Human Natural Killer (NK) Cell Growth

The NK-92MI cell line (CRL-2408, ATCC, USA) was diluted to 2 × 10^7^ viable cells/mL using 2 mM l-glutamine, 0.2 mM myoinositol, 20 mM folic acid, 0.1 mM 2-mercaptoethanol, 12.5% fetal bovine serum (FBS) and 12.5% horse serum (Myelocult) in α-MEM medium. Cell proliferation during culture inT-25 flasks in the presence of samples at a concentration of 0.5 g/L was determined. The cells cultures were then centrifuged to obtain the supernatant. 900 L of the NK-92MI cell line was aliquoted into 24 well plates at 4~5 × 10^4^ viable cells/mL, and 100 L of the supernatant from T cells was then placed into the well and cultured for 48 h. The growth of NK-92MI cells was measured over six days using a cell counter [60,61].

### 3.7. HPLC Analysis

The extracts of *S. maxima* grown in seawater or deep-sea water were analyzed by HPLC (Hewlett Packard HP1100, USA) after filtering with 4.5 m filter paper. The samples were separated at 27 °C on Phenomenex (250 × 4.60 mm, 5 micron) column. The mobile phase consisted of acetone, acetonitrile, and orthophosphoric acid in the ratio of 70:20:10 and a flow rate of 1 mL/min was used. The HPLC peaks were monitored at 254 nm for 30 min and β-carotene, ascorbic acid, and thiamin were used as standards to assess the retention time of these compounds under the same conditions [62].

### 3.8. Statistical Analysis

All experiments were conducted in three duplicates, and expressed as mean ± S.D. Data were analyzed using the Statistical Analysis System software. Fisher’s Least Significant Difference (LSD) was used to determine significant difference among the treatments at *p* < 0.05.

## 4. Conclusions

There have been several promising results of having better cell growth and physiological performance of micro algae grown in deep-sea water; however, there are very few data related to the biological functions of the extracts, as well as a lack of scientific explanation. In this work, it was clearly shown that the amounts of β-Carotene and ascorbic acid of the extract were significantly increased by cultivating *S. maxima* in deep-sea water, possibly due to rich important minerals for algal cell growth and relatively low pathogens. It was interesting that the extracts grown from deep-sea water (DSW) had less cytotoxicity and better anticancer activities than those from conventional seawater (SW), which would imply that the mineral compositions of deep-sea water plays a role in constituting cell materials to affect the immune functions. The selective anticancer activities of DSW could be caused by improving immuno-modulatory activities associated with the increase of key cytokines, TNF-α and IL-2. Our work demonstrates that DSW increased TRAIL-induced apoptosis, which can sensitize several cancer cells, by the result that Bcl-2 expression level was significantly decreased in the presence of the DSW extracts. We believe this is the first report that *S. maxima* grown in deep-sea water has better immune activation activities than that in surface seawater because the extracts contained high amounts of β-carotene and ascorbic acid, even though new biologically active components in the extract should be investigated.

## Figures and Tables

**Table 1:** Inhibition of cell growth of MCF-7 and Hep3B cancer cell lines after addition of the extract under various cultivation conditions.

Concentration (mg/mL)	Seawater (SW)		Deep-sea water (DSW)	
	
	MCF-7	Hep3B	MCF-7	Hep3B
0.2	43.5 ± 0.22	44.2 ± 0.22 ***	46.5 ± 0.23 ***	45.3 ± 0.40 **
0.4	52.1 ± 0.23 *	55.6 ± 0.24	56.1 ± 0.43	59.4 ± 0.47
0.6	61.7 ± 0.20	62.4 ± 0.23 *	68.2 ± 0.26 **	74.4 ± 0.44 *
0.8	73.4 ± 0.27 **	75.1 ± 0.28	77.5 ± 0.39	82.1 ± 0.41 ***
1.0	80.5 ± 0.41 ***	83.4 ± 0.31 **	89.6 ± 0.42 *	93.5 ± 0.43 **

Mean ± SD from triplicate separated experiments are shown. Each value were compared with control at

* *p* < 0.01,

** *p* < 0.005,

*** *p* < 0.001 by Student *t*-test.

**Table 2:** Cell growth and IL-6 and Tumor Nucreosis Factor-α (TNF-α) secretion from human immune T cells after adding 1.0 mg/mL of *S. maxima* extracted under various cultivation conditions.

Sample	Cultivation time (Day)	T cell			B cell		
	
		Viable cell density (×10^4^ cells/mL)	Cytokine secretion (×10^−4^ pg/cell)		Viable cell density (×10^4^ cells/mL)	Cytokine secretion (×10^−4^ pg/cell)	
	
			IL-6	TNF-α		IL-6	TNF-α
DSW	1	2.9 ± 0.02 *	1.2 ± 0.16 ***	1.3 ± 0.11 *	2.8 ± 0.07 **	1.3 ± 0.01 **	1.4 ± 0.06 *
	2	4.2 ± 0.04 **	1.6 ± 0.07 ***	1.5 ± 0.03 ***	3.9 ± 0.06 *	1.8 ± 0.15 ***	1.7 ± 0.04 ***
	3	6.5 ± 0.05 *	3.2 ± 0.06 **	2.3 ± 0.07 **	5.8 ± 0.02 **	3.4 ± 0.23 **	2.6 ± 0.01 ***
	4	8.8 ± 0.06 ***	3.7 ± 0.03 *	3.3 ± 0.05 *	7.6 ± 0.10 *	3.6 ± 0.16 *	3.4 ± 0.08 *
	5	16.0 ± 0.08 ***	5.3 ± 0.04 ***	4.8 ± 0.15 **	12.0 ± 0.06 *	4.0 ± 0.07 **	5 ± 0.02 **
	6	24.0 ± 0.09 *	6.4 ± 0.11 *	5.6 ± 0.11 *	18.0 ± 0.08 ***	5.9 ± 0.18 **	5.7 ± 0.13 *

SW	1	3.1 ± 0.06 *	1.1 ± 0.15 ***	1.3 ± 0.16 **	2.9 ± 0.03 ***	0.9 ± 0.12 **	1.1 ± 0.15 *
	2	3.8 ± 0.09 ***	1.6 ± 0.20 *	1.7 ± 0.03 ***	3.6 ± 0.05 *	1.3 ± 0.09 *	1.9 ± 0.06 **
	3	5.4 ± 0.07 *	2.6 ± 0.18 *	2.6 ± 0.04 *	5.1 ± 0.02 *	2.4 ± 0.16 *	2.6 ± 0.03 **
	4	7.3 ± 0.05 ***	3.2 ± 0.07 ***	3.7 ± 0.01 ***	7.0 ± 0.12 ***	3 ± 0.13 ***	3.8 ± 0.11 ***
	5	9.6 ± 0.10 *	3.8 ± 0.06 *	4.3 ± 0.20 *	9.2 ± 0.09 *	3.7 ± 0.02 **	4.2 ± 0.13 *
	6	11.2 ± 0.09 **	5.3 ± 0.04 *	5.6 ± 0.18	11.0 ± 0.08 **	5.6 ± 0.05 *	5.7 ± 0.13 ***

Mean ± SD from triplicate separated experiments are shown. Each value were compared with control at

* *p* < 0.01,

** *p* < 0.005,

*** *p* < 0.001 by Student *t*-test.

(SW: seawater, DSW: deep-sea water).

**Table 3:** Comparison of the mineral compositions of deep-sea water and seawater.

Composition		Sea water	Deep seawater
	
		mg/kg	mg/kg
Main element	K	-	380
	Na	8900	10780
	Ca	393	403
	Mg	1080	1320
	SiO	2.0	2.8
	Cl^−^	12000	19350
	SO_4_^−2^	2648	898
	F^−^	1	1.3
	Li	0.16	0.18
	Sr	7.8	14.3

Trace element	Fe	0.02	0.09
	Mn	0.03	0.16
	Cu	-	0.26
	Pb	-	0.11
	Zn	-	0.45
	Cd	-	0.05
	Ni	-	0.36

Nutritive salts	NO^3−^	0.04	0.28
	PO_4_^3−^	0.012	0.06
	Si	0.44	2.8
